# Supplementary material for: The Impact of Dance Movement Interventions on Psychological Health in Older Adults without Dementia: A Systematic Review and Meta-Analysis
Source: Brain Sci. 2023 Jun 22;13(7):981. doi: 10.3390/brainsci13070981 (PMC10377702; doi:10.3390/brainsci13070981)
Supplement: Supplementary file 1 [file brainsci-13-00981-s001.zip › brainsci-2441456-supplementary author sent.pdf]

The impact of dance movement interventions on psychological health in older adults without dementia: A systematic review and meta-analysis

Supplementary Material

Contents

1 PRISMA Checklist 2
2 Search Terms 5
2.1 Pubmed 5
2.2 Web of Science 5
2.3 PsycInfo (via EBSCO) 5
3 Detailed Characteristics of Interventions 7
4 Detailed Outcome Measures 10
5 Risk of Bias Rating 14
6 Statistical Analyses and Formulas 15
7 Publication Bias 16
8 Forest Plots 17
8.1 Overall psychological health 17
8.2 Quality of Life 18
8.3 General cognitive function 19
9 Further Analyses 19
9.1 Outlier Analysis 19

1 PRISMA Checklist

Section and Topic	Item #	Checklist item	Location where item is reported
**TITLE**			
Title	1	Identify the report as a systematic review.	1
**ABSTRACT**			
Abstract	2	See the PRISMA 2020 for Abstracts checklist.	1
**INTRODUCTION**			
Rationale	3	Describe the rationale for the review in the context of existing knowledge.	1-3
Objectives	4	Provide an explicit statement of the objective(s) or question(s) the review addresses.	3
**METHODS**			
Eligibility criteria	5	Specify the inclusion and exclusion criteria for the review and how studies were grouped for the syntheses.	3-4
Information sources	6	Specify all databases, registers, websites, organisations, reference lists and other sources searched or consulted to identify studies. Specify the date when each source was last searched or consulted.	4
Search strategy	7	Present the full search strategies for all databases, registers and websites, including any filters and limits used.	4 and Supplement
Selection process	8	Specify the methods used to decide whether a study met the inclusion criteria of the review, including how many reviewers screened each record and each report retrieved, whether they worked independently, and if applicable, details of automation tools used in the process.	4-5
Data collection process	9	Specify the methods used to collect data from reports, including how many reviewers collected data from each report, whether they worked independently, any processes for obtaining or confirming data from study investigators, and if applicable, details of automation tools used in the process.	4-5
Data items	10a	List and define all outcomes for which data were sought. Specify whether all results that were compatible with each outcome domain in each study were sought (e.g. for all measures, time points, analyses), and if not, the methods used to decide which results to collect.	4 and Supplement
	10b	List and define all other variables for which data were sought (e.g. participant and intervention characteristics, funding sources). Describe any assumptions made about any missing or unclear information.	5
Study risk of bias assessment	11	Specify the methods used to assess risk of bias in the included studies, including details of the tool(s) used, how many reviewers assessed each study and whether they worked independently, and if applicable, details of automation tools used in the process.	5
Effect measures	12	Specify for each outcome the effect measure(s) (e.g. risk ratio, mean difference) used in the synthesis or presentation of results.	5 and supplement
Synthesis methods	13a	Describe the processes used to decide which studies were eligible for each synthesis (e.g. tabulating the study intervention characteristics and comparing against the planned groups for each synthesis (item #5)).	5-6
	13b	Describe any methods required to prepare the data for presentation or synthesis, such as handling of missing summary statistics, or data conversions.	5-6

Section and Topic	Item #	Checklist item	Location where item is reported
	13c	Describe any methods used to tabulate or visually display results of individual studies and syntheses.	5
	13d	Describe any methods used to synthesize results and provide a rationale for the choice(s). If meta-analysis was performed, describe the model(s), method(s) to identify the presence and extent of statistical heterogeneity, and software package(s) used.	5-6 and Supplement
	13e	Describe any methods used to explore possible causes of heterogeneity among study results (e.g. subgroup analysis, meta-regression).	5
	13f	Describe any sensitivity analyses conducted to assess robustness of the synthesized results.	5
Reporting bias assessment	14	Describe any methods used to assess risk of bias due to missing results in a synthesis (arising from reporting biases).	5
Certainty assessment	15	Describe any methods used to assess certainty (or confidence) in the body of evidence for an outcome.	n.a.
**RESULTS**			
Study selection	16a	Describe the results of the search and selection process, from the number of records identified in the search to the number of studies included in the review, ideally using a flow diagram.	6-7
	16b	Cite studies that might appear to meet the inclusion criteria, but which were excluded, and explain why they were excluded.	n.a.
Study characteristics	17	Cite each included study and present its characteristics.	Table 1 and Supplement
Risk of bias in studies	18	Present assessments of risk of bias for each included study.	Supplement
Results of individual studies	19	For all outcomes, present, for each study: (a) summary statistics for each group (where appropriate) and (b) an effect estimate and its precision (e.g. confidence/credible interval), ideally using structured tables or plots.	Supplement
Results of syntheses	20a	For each synthesis, briefly summarise the characteristics and risk of bias among contributing studies.	Figure 2; 11
	20b	Present results of all statistical syntheses conducted. If meta-analysis was done, present for each the summary estimate and its precision (e.g. confidence/credible interval) and measures of statistical heterogeneity. If comparing groups, describe the direction of the effect.	Table 2; 12
	20c	Present results of all investigations of possible causes of heterogeneity among study results.	12-13
	20d	Present results of all sensitivity analyses conducted to assess the robustness of the synthesized results.	12-13
Reporting biases	21	Present assessments of risk of bias due to missing results (arising from reporting biases) for each synthesis assessed.	n.a.
Certainty of evidence	22	Present assessments of certainty (or confidence) in the body of evidence for each outcome assessed.	n.a.
**DISCUSSION**			
Discussion	23a	Provide a general interpretation of the results in the context of other evidence.	13-15
	23b	Discuss any limitations of the evidence included in the review.	16
	23c	Discuss any limitations of the review processes used.	16

Section and Topic	Item #	Checklist item	Location where item is reported
	23d	Discuss implications of the results for practice, policy, and future research.	14-15
**OTHER INFORMATION**			
Registration and protocol	24a	Provide registration information for the review, including register name and registration number, or state that the review was not registered.	1; 3
	24b	Indicate where the review protocol can be accessed, or state that a protocol was not prepared.	3
	24c	Describe and explain any amendments to information provided at registration or in the protocol.	n.a.
Support	25	Describe sources of financial or non-financial support for the review, and the role of the funders or sponsors in the review.	16
Competing interests	26	Declare any competing interests of review authors.	17
Availability of data, code and other materials	27	Report which of the following are publicly available and where they can be found: template data collection forms; data extracted from included studies; data used for all analyses; analytic code; any other materials used in the review.	16

2 Search Terms

2.1 Pubmed

1	dance* OR dancing* OR dancing [MeSH Terms] OR dance therapy [MeSH Terms] OR "ballet" OR "jazz" OR "hiphop" OR "salsa" OR "zumba" OR "tango" OR "eurhythmics"
2	"music" AND "exercise"
3	aged [MeSH Terms] OR "aging" OR "ageing" OR "senior" OR "elderly" OR "older adults" OR "older people" OR "cognitive impairment" OR "cognitive decline"
4	psychological* OR neuropsychological* OR "mental health" OR "quality of life" OR "wellbeing" OR "life satisfaction" OR "mood"
5	random* OR control* OR "clinical trial"
6	1 OR 2 AND 3 AND 4 AND 5

2.2 Web of Science

1	TS=(dance* OR dancing* OR dancing OR dance therapy OR "ballet" OR "jazz" OR "hiphop" OR "salsa" OR "zumba" OR "tango" OR "eurhythmics")
2	TS=(aged OR "aging" OR "ageing" OR "senior" OR "elderly" OR "older adults" OR "older people" OR "cognitive impairment" OR "cognitive decline")
3	TS=(psychological* OR neuropsychological* OR "mental health" OR "quality of life" OR "wellbeing" OR "life satisfaction" OR "mood")
4	TS=(random* OR control* OR "clinical trial")
5	1 AND 2 AND 3 AND 5

2.3 PsycInfo (via EBSCO)

1	dance* OR dancing* OR dancing OR dance therapy [MeSH Terms] OR "ballet" OR "jazz" OR "hiphop" OR "salsa" OR "zumba" OR "tango" OR "eurhythmics"
2	"music" AND "exercise"
3	aged [MeSH Terms] OR "aging" OR "ageing" OR "senior" OR "elderly" OR "older adults" OR "older people" OR "cognitive impairment" OR "cognitive decline"

4	psychological* OR neuropsychological* OR "mental health" OR "quality of life" OR "wellbeing" OR "life satisfaction" OR “mood”
5	random* OR control* OR “clinical trial”
6	1 OR 2 AND 3 AND 4 AND 5

3 Detailed Characteristics of Interventions

Table S1. Characteristics of Dance Movement Interventions (DMI) and control conditions.

Study	DMI								Control Intervention	
	Type of DMI	Description	Period and Frequencies	Qualification of instructor	Manual	Setting	Type of Music/ Rhythm	Adherence	Control Type(s)	Description
Alves, 2013 [66]	Ballroom Dance	Groups learned dance sequences of different rhythms led by certified instructor; warm-up and cool-down	16 weeks, 2/week, 120 min	Certified dance instructor	NR	Group setting	e.g. Samba, Bolero	90%	AC: Walking<br>PC: no contact	Walking in a group matched to activity level of DMI group, passive control
Bisbe et al., 2020 [75]	Choreo-graphy	Performing aerobic dances in groups of 8 in light to moderate intensity led by physical therapist and videos for visual support	12 weeks, 2/week, 60 min	Physical Therapist specialized in geriatrics	yes	Day care hospital, group setting	Salsa, Rock, Jive, Pop	95%	AC: Physical Therapy	Multimodal physical therapy program (strength, gait, flexibility, balance training)
Chang et al., 2021 [73]	Square Dance	Chinese square dance performed outdoors in groups with practice videos before intervention for familiarization	18 weeks, 3/week, 30 min	National social sports instructors	NR	Group setting, outdoor	Dance music with simple melodies	88%	PC: Usual Care	Liberal daily lifestyle
Cruz-Ferreira et al., 2015 [76]	Creative Dance	Five elements of movement: body, space, time, dynamic and relationship, performed in groups	24 weeks, 2/week, 50 min	Nurse, dance teacher	NR	Local health center, group setting	differed to theme (e.g. classic, jazz, pop, ethnics)	85%	PC: Waitlist	NA
Esmail et al., 2020 [71]	Dance Movement	ADTA-based program adapted to older adults	12 weeks, 3/week, 60 min	Registered ADTA therapist	Yes	Gym facility, group setting	different styles	91%	AC: Aerobic Exercise Training<br>PC: Waitlist	Cardio-vascular training on bicycle
Eyigor et al., 2009 [77]	Folkloric Dance	Dancing folkloric routines in groups led by dance senior, additional instruction to walk for 30 minutes twice a week	8 weeks, 3/week, 60 min	folklore dance expert	NR	Reha-bilitation unit, group setting	Turkish folk music	NR	PC: no intervention	NA

Study	DMI								Control Intervention	
	Type of DMI	Description	Period and Frequencies	Qualification of instructor	Manual	Setting	Type of Music/ Rhythm	Adherence	Control Type(s)	Description
Hars et al., 2014 [67]	Eurythmy	Multitask exercises of progressive difficulty with different objectives in a group	25 weeks, 1/week, 60 min	Experienced instructor	Yes	Group setting	Piano music	79%	PC: Waitlist	NA
Hui et al., 2009 [70]	Aerobic Dance	Low impact dancing choreography consisting of cross steps and cha-cha steps in a group created by professional dance instructor and physical therapist	12 weeks, 2/week, 50 min	Professional dance instructor, physical therapist	NR	Group setting	Canto-Pop Song	92%	PC: no intervention	NA
Kosmat & Vranic, 2017 [74]	Standard Dance	dancing/ learning slow waltz under supervision of trained dance pedagogist	10 weeks, 1/week, 45 min	Trained dance teacher	NR	Care center, group setting	Waltz	NR	AC: Social Discussion	Various topics (needs and interests of participants) discussed in groups
Lazarou et al., 2017 [78]	Ballroom Dance	Learning several ballroom dances with routines and music led by experienced dance instructor	40 weeks, 2/week, 60 min	Dance instructor	NR	Group setting	Tango, Waltz, Rumba, Chachacha, Swing Salsa, etc.	NR	PC: no intervention	NA
Liao et al., 2018 [69]	Music and Tai-Chi (Combined)	Combination of 24 Tai Chi movements and music in groups	12 weeks, 3/week, 50 min	NR	NR	Group setting	Soft Chinese folk music	NR	AC: Routine health education	NR
Mishra et al., 2022 [79]	Folkloric Dance	Indian Folk Dance Therapy performed in groups in moderate intensity with programmed choreographies	6 weeks, 5/week, 60min	NR	NR	Group setting	Indian folk music	NR	AC: exercise program	Conventional physical exercises according to American College of

Study	DMI								Control Intervention	
	**Type of DMI**	**Description**	**Period and Frequencies**	**Qualification of instructor**	**Manual**	**Setting**	**Type of Music/ Rhythm**	**Adherence**	**Control Type(s)**	**Description**
										Sports guidelines
Serrano-Guzmán et al., 2016 [80]	Dance Therapy (Flamenco)	Low-impact aerobics and stretching mixed with dance movements based on flamenco performed in groups	8 weeks, 3/week, 50 min	NR	Yes	Group setting	Flamenco	100%	AC: Self-care treatment advice	Follow physical activity recom-mendations
Zhu et al., 2018 [72]	Aerobic Dance	Learning dance routine in groups together with dance instructor	12 weeks, 3/week, 35 min	Dance instructor, physical therapist	NR	Group setting	Musical phrases	Median attended sessions: 36	PC: no intervention	NA

Key: DMI, Dance Movement Intervention; AC, Active Control; PC, Passive Control; NR, Not reported; NA, Not applicable, ADTA, American Dance Therapy Association.

4 Detailed Outcome Measures

Table S2. Overview of outcome measures according to constructs of psychological health and Quality of Life.

Study	Assessment tool	Construct					
		Psychological health	Quality of Life	Positive domain		Negative domain	
				Wellbeing	Social integration	Anxiety/ Stress	Depression
Alves, 2013 [66]	Ryff's PWBS - Autonomy	♦		♦			
	Ryff's PWBS - Environmental Mastery	♦		♦			
	Ryff's PWBS - Personal Growth	♦		♦			
	Ryff's PWBS - Positive Relations with others	♦			♦		
	Ryff's PWBS - Purpose in life	♦		♦			
	Ryff's PWBS - Self Acceptance	♦		♦			
	Pittsburgh Sleep Quality Index (PSQI)		♦				
	Beck Anxiety Inventory (BAI)	♦				♦	
	Perceived Stress Scale (PSS)	♦				♦	
Bisbe et al., 2020 [75]	SF-36 - Total Score	♦	♦				
	Hospital Anxiety and Depression Scale – Anxiety (HADS-A)	♦				♦	

Study	Assessment tool	Construct					
		Psychological health	Quality of Life	Positive domain		Negative domain	
				Wellbeing	Social integration	Anxiety/ Stress	Depression
	Hospital Anxiety and Depression Scale – Depression (HADS-D)	♦					♦
Chang et al., 2021 [73]	SF-12 - Mental Component	♦	♦	♦			
	Geriatric Depression Scale -15 (GDS-15)	♦					♦
Cruz-Ferreira et al., 2015 [76]	Life Satisfaction Scale (LSS)	♦		♦			
Esmail et al., 2020 [71]	SF-12 - Mental Component	♦	♦	♦			
	Health Promoting Lifestyle Profile-2 (HPLP2)		♦				
	Mental Health Continuum Short Form (MHC)	♦	♦	♦			
	Lubben Social Network Scale Revised (LSNS)	♦			♦		
	State-Trait Anxiety Inventory – Trait (STAI-Trait)	♦				♦	
	Brief Pain Inventory (BPI)		♦				
Eyigor et al., 2009 [77]	SF-36 - Pain		♦				
	SF-36 - Social Functioning	♦	♦		♦		
	SF-36 - Role Emotional	♦	♦	♦			

Study	Assessment tool	Construct					
		Psychological health	Quality of Life	Positive domain		Negative domain	
				Wellbeing	Social integration	Anxiety/ Stress	Depression
	SF-36 - Mental Health	♦	♦	♦			
	SF-36 - Physical Functioning		♦				
	SF-36 - Role Physical		♦				
	SF-36 - General Health		♦				
	SF-36 - Vitality	♦	♦	♦			
Hars et al., 2014 [67]	Hospital Anxiety and Depression Scale – Anxiety (HADS-A)	♦				♦	
	Hospital Anxiety and Depression Scale – Depression (HADS-B)	♦					♦
Hui et al., 2009 [70]	SF-36 - General Health		♦				
	SF-36 - Bodily Pain		♦				
	SF-36 - Role of Emotion	♦	♦	♦			
Kosmat & Vranic, 2017 [74]	Satisfaction with Life Scale (SWLS)	♦		♦			
	General Self Efficacy Scale (GSE)	♦					

Study	Assessment tool	Construct					
		Psychological health	Quality of Life	Positive domain		Negative domain	
				Wellbeing	Social integration	Anxiety/ Stress	Depression
Lazarou et al., 2017 [78]	Neuropsychiatric Inventory (NPI)	♦					
Liao et al., 2018 [69]	Geriatric Depression Scale (GDS)	♦					♦
Mishra et al., 2022 [79]	SF-36 - Mental Component	♦	♦	♦			
	SF-36 - Physical Component		♦				
Serrano-Guzmán et al., 2016 [80]	SF-12 - Mental Component	♦	♦	♦			
Zhu et al., 2018 [72]	Geriatric Depression Scale – 15 (GDS-15)	♦					♦
	SF-36	♦	♦				

Key: PWBS, Psychological Well-Being Scales; SF-36, 36-Item Short Form Health Survey; SF-12, 12-Item Short Form Health Survey.

5 Risk of Bias Rating

Methodological quality of each study was assessed with the Risk of Bias (RoB) Tool 2 [54]. Resulting ratings for individual studies according to domains assessed can be seen in Figure S1. Each study was rated regarding an intention-to-treat approach. Detailed information on each domain is given below.

Figure S1. Risk of Bias rating for individual primary studies.

Study ID	Experimental	Comparator	D1	D2	D3	D4	D5	Overall
Alves, 2013	Ballroom Dance	No-contact-control	+	-	-	!	-	-
Bisbe et al., 2020	Choreography Training	Physical Therapy	!	!	+	+	+	!
Chang et al., 2021	Square Dance Exercise	Usual Care	!	!	+	+	+	!
Cruz-Ferreira et al., 2015	Creative Dance	Waitlist	+	+	+	-	+	-
Esmail et al., 2020	Dance Movement Training	Waitlist	+	!	+	!	-	-
Eyigor et al., 2009	Folkloric Dance	Usual Care	!	!	+	-	-	-
Hars et al., 2014	Eurhtyhms	Waitlist	+	+	+	-	+	-
Hui et al., 2009	Aerobic Dance	Usual Care	-	!	!	-	-	-
Kosmat & Vranic, 2017	Standard Dance	Guided discussion	!	!	-	!	!	-
Lazarou et al., 2017	Balroom Dance	Usual Care	+	-	+	+	-	-
Liao et al., 2018	Tai Chi with Music	Routine Health Education	!	!	+	+	+	!
Mishra et al., 2022	Folkloric Dance	Exercise Program	!	+	+	!	+	!
Serrano-Guzmán et al., 2016	Dance Therapy	Self-Care Advice	+	+	+	!	+	!
Zhu et al., 2018	Aerobic Dance Routine	Usual care	+	!	+	-	+	-

+ Low risk
! Some concerns
- High risk

D1 Randomisation process
D2 Deviations from the intended interventions
D3 Missing outcome data
D4 Measurement of the outcome
D5 Selection of the reported result

Note. Risk of Bias was assessed with the RoB tool 2 regarding an intention-to-treat methodology following the five domains (D1-D5).

The rating procedures yielded following results:

- **"Randomization process" (D1) bias rating:** A large proportion of studies was rated as being at "low risk" (k = 7, 50%) or "some concerns" (k = 6, 43%). Individual studies confirmed a successful randomization process by showing no significant differences in baseline measures between groups.
- "**Deviations from the intended interventions" (D2) bias rating:** Eight studies (57%) were rated with "some concerns" and two studies (14%) were rated at "high risk". "High" bias ratings reflected the use of per-protocol analysis (k = 8; 57%) and bias for blinding (k = 4, 29%) as inherent to non-pharmacological interventions. Blinding of participants is barely possible due to the nature of DMI. Blinding of study assessors was reported in four studies (29%). Psychological outcomes were assessed via patient/participant-reported outcomes, which could be biased. Four studies (29%) applied an intention-to-treat analysis and were rated at "low risk".

- **"Missing outcome data" (D3) bias rating:** A large proportion of studies (*k* = 11, 79%) was assigned to "low risk" of bias. One study reported a high drop-out rate for only one group which resulted in an unbalanced group size included in the statistical analyses [66] and was therefore rated as being at high risk. The remaining two studies did not provide sufficient information on how missing outcome data occurred and was handled.
- **"Measurement of the outcome" (D4) bias rating:** Five studies (36%) were rated with "some concerns" and five studies (36%) were rated at "high risk". Risk of bias was seen in the self-reported assessment of psychological health and participants' awareness of group allocation especially with passive controls.
- **"Selection of reported outcomes" (D5) bias rating:** Ratings were split between studies rated at "low risk" (*k* =8, 57%) or studies rated as "high risk" (*k* =5, 36%). Risk of bias was seen for studies that reported selected psychological health outcomes in the result sections.

6 Statistical Analyses and Formulas

The calculations described below were applied and implemented using the free statistical computation software R (version 4.0.3) for each outcome score to determine effect sizes. Firstly, the standardized mean change within each study arm (DMI and control condition) between pre- to post-intervention scores was calculated with the "metafor" package (version 3.0-2) in R [65]. Secondly, the difference between these change scores and sampling variance was determined. Following Morris [59], this score was then divided by the pooled pre-test standard deviations and a bias correction factor was applied resulting in the effect size g (formula given below). The formula of the present effect size calculation involves knowledge on pre-test post-test correlations, which are rarely reported. A value of r = 0.50 was employed as a common value for substitution of unknown correlations [108].

Effect size formula (see equation 8 in Morris [59]):

$$d_{ppc2} = c_P \left[\frac{\left(M_{post,T} - M_{pre,T}\right) - \left(M_{post,C} - M_{pre,C}\right)}{SD_{pre}} \right]$$

with the pooled standard deviation (see equation 9):

$$SD_{pre} = \sqrt{\frac{(n_T - 1)SD_{pre,T}^2 + (n_C - 1)SD_{pre,C}^2}{n_T - n_C - 2}}$$

and the bias correction factor was defined according to equation 6e in Hedges [109]:

$$c(m) = \frac{\Gamma\left(\frac{m}{2}\right)}{\sqrt{\frac{m}{2}}\,\Gamma\left(\frac{m-1}{2}\right)}$$

with: $$m = n_T - n_C - 2$$

the variance of effect size was calculated according to equation 13 in Becker [110]:

$$var = 2(1-r)\left(\frac{1}{n_T} + \frac{1}{n_C}\right) + \frac{\Delta^2}{2(n_T + n_C)}$$

Operator	Definition
M	Mean score of a test outcome
T	Treatment group
C	Control group
Post	Immediately post-intervention
Pre	prior to intervention (baseline assessment)
n	Sample size of group
r	Correlation between M_{pre} and M_{post}
C_p	Bias correction factor
SD	Standard Deviation
△	Effect Size

7 Publication Bias

Figure S2. Funnel Plot.

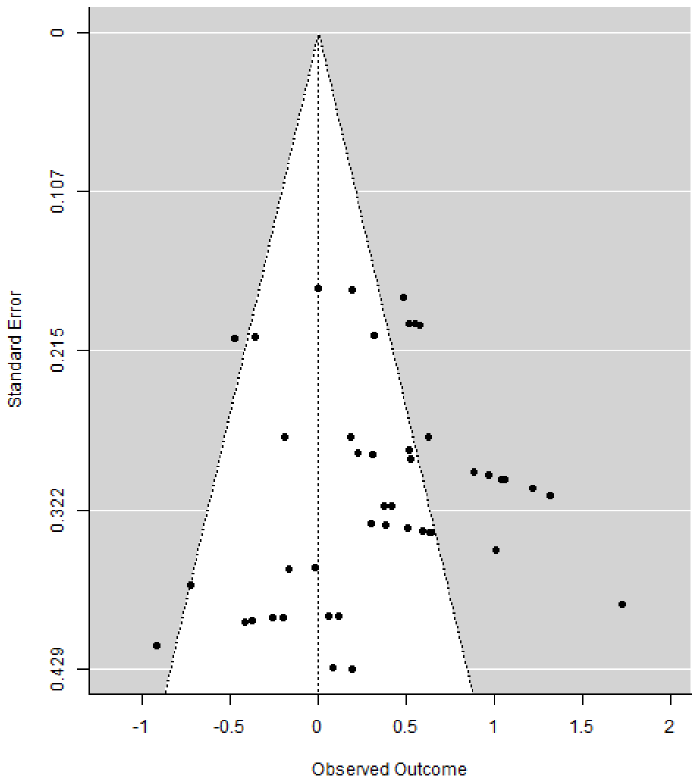

Note. Observed effect sizes per individual outcome measure included in data synthesis were plotted against standard error. Assessment of asymmetry was confirmed with an Egger's regression test indicating no evidence for publication bias.

8 Forest Plots

8.1 Overall psychological health

Figure S3. Forest Plot for psychological health.

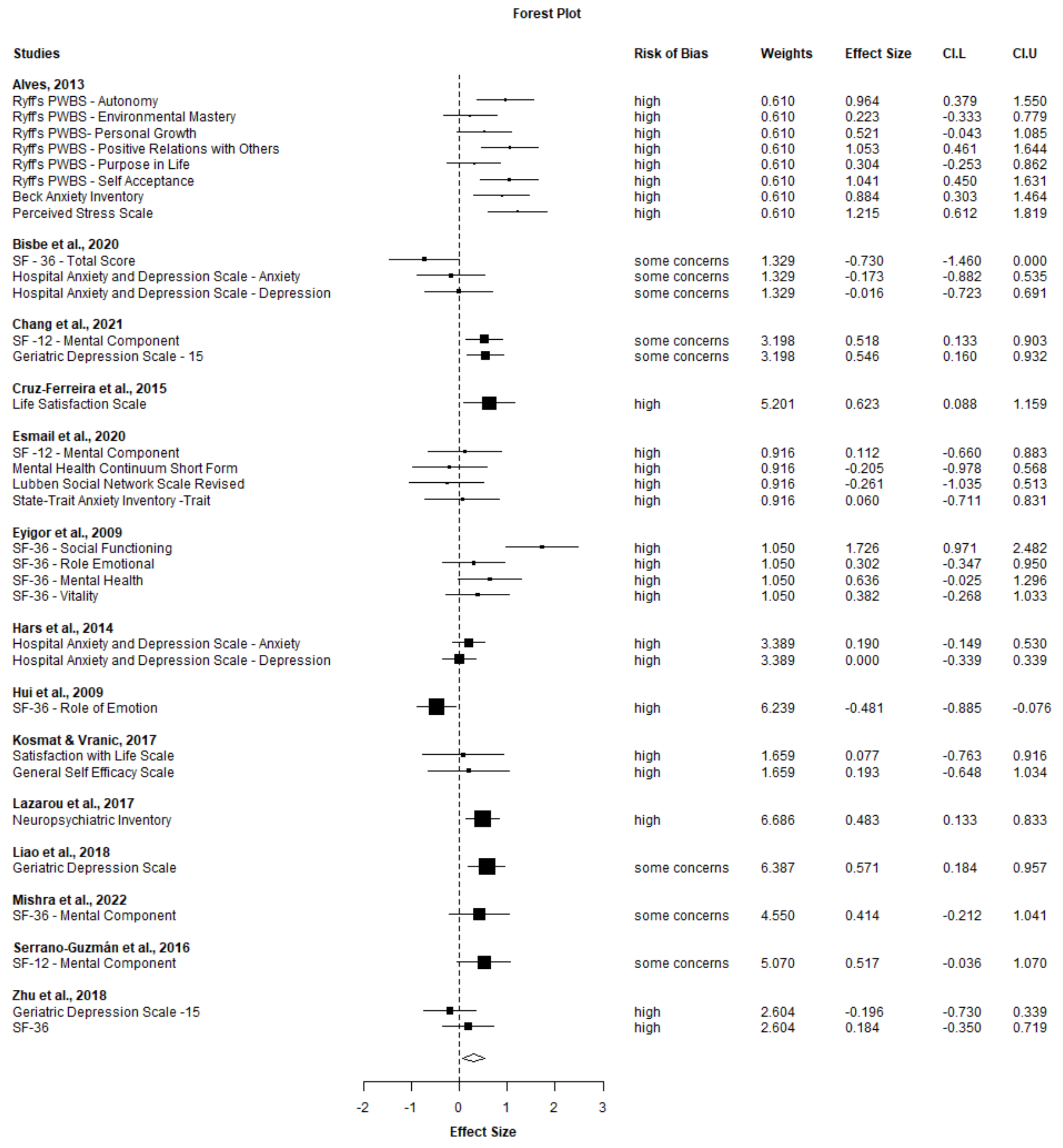

Note. Effect sizes are presented for each study according to the assessment tools used, respectively. Values on the left side of the null line favor control condition and values on the right side favor DMI. Effect sizes of DMI compared to passive control conditions were included where applicable for individual studies (otherwise active control groups were used as comparator). Key: CI.L, Confidence interval lower bound; CI.U, Confidence interval upper bound, Ryff's PWBS, Ryff's Psychological Wellbeing Scales; SF-36, 36-Item Short Form Health Survey; SF-12, 12-Item Short Form Health Survey.

8.2 Quality of Life

Figure S4. Forest Plot for Quality of Life.

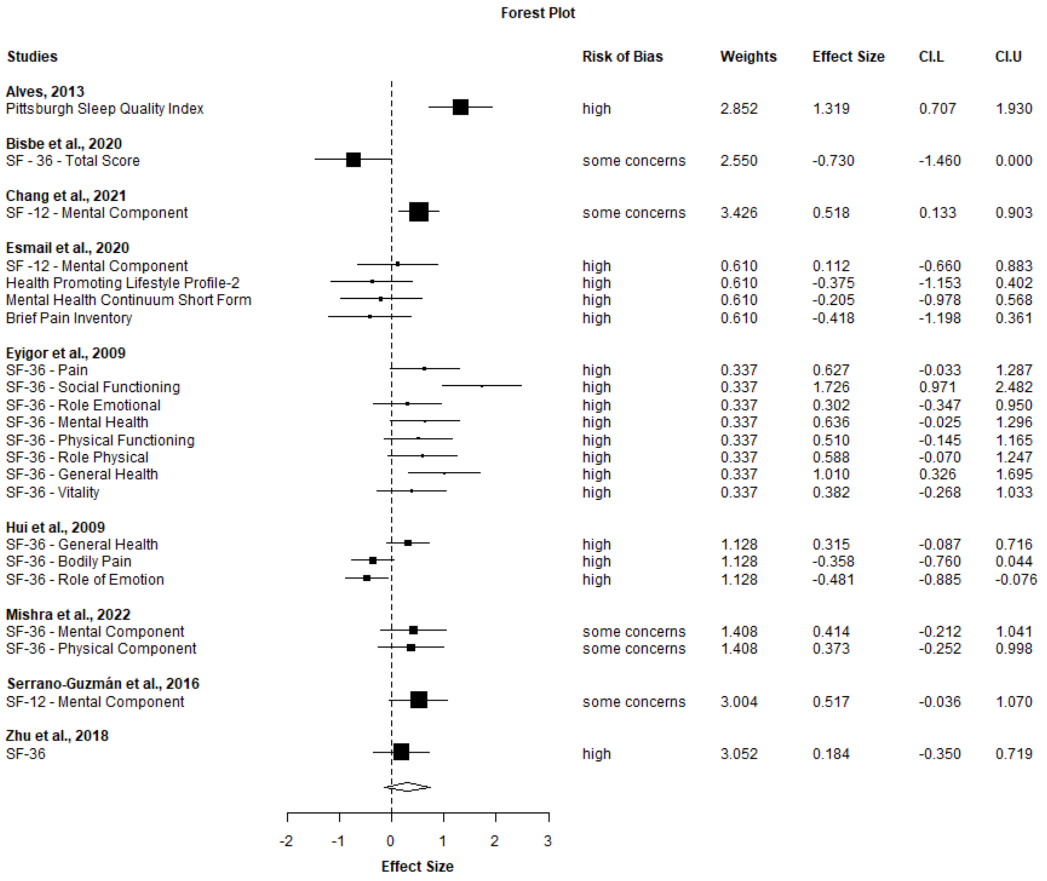

Note. Effect sizes are presented for each study according to the assessment tools used, respectively. Values on the left side of the null line favor control condition and values on the right side favor DMI. Effect sizes of DMI compared to passive control conditions were included where applicable for individual studies (otherwise active control groups were used as comparator). Key: CI.L, Confidence interval lower bound; CI.U, Confidence interval upper bound, SF-36, 36-Item Short Form Health Survey; SF-12, 12-Item Short Form Health Survey.

8.3 General cognitive function

Figure S5. Forest Plot for general cognitive function.

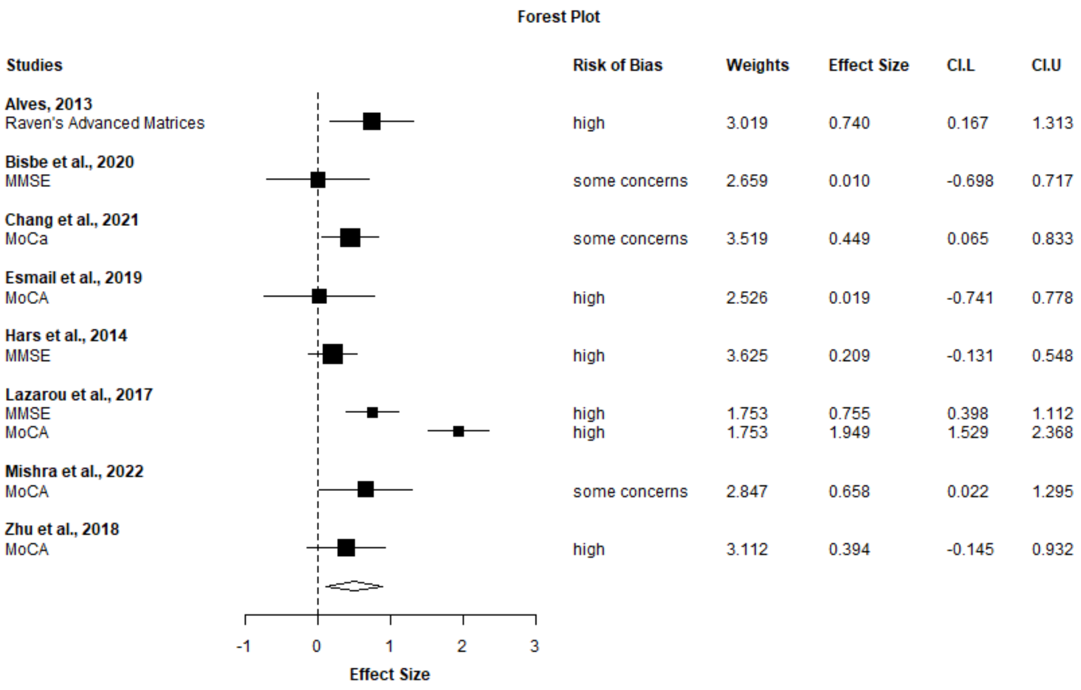

Note. Effect sizes are presented for each study according to assessment tools of general cognitive function, respectively. Values on the left side of the null line favor control condition and values on the right side favor DMI. Effect sizes of DMI compared to passive control conditions were included where applicable for individual studies (otherwise active control groups were used as comparator). Key: CI.L, Confidence interval lower bound; CI.U, Confidence interval upper bound; MMSE, Mini-Mental State Examination; MOCA, Montreal Cognitive Assessment.

9 Further Analyses

9.1 Outlier Analysis

Post-hoc inspection of studies with regard to possible outliers resulted in the identification of six individual scales. Following scales were excluded as their particular CIs did not overlap with the overall effect CI:

- Pittsburgh Sleep Quality Index; Perceived Stress Scale [66]
- 36-Short Form [75]
- SF-36 Social Functioning [77]
- SF-36 Bodily Pain; SF-36 Role of Emotion [70]

Analyses were repeated as described in the method section with results provided below (see Table S3). Lastly, exclusion of the study by Hui and colleagues [70], due to methodological considerations

concerning the randomization process, substantiated our findings for overall psychological health (g = 0.37; CI 95% [0.21, 0.54]; *p* = 0.0006; I^2 = 18.36).

Table S3. Results of the meta-analysis for psychological health outcomes and Quality of Life with outliers removed.

	**Construct**	**k *(N* ES)**	**ES**	**95% CI**	**SE**	**df**	***p*-value**	**I^2 %**
Overall Psychological health	all combined	13 (29)	0.37	[0.21, 0.54]	0.07	12	.0006	18.36
Positive domain	Wellbeing	8 (15)	0.46	[0.32, 0.61]	0.06	6	.0003	0
Negative domain	Anxiety/Stress	4 (4)	0.27	[-0.44, 0.97]	0.21	3*	*	52.28
	Depression	5 (5)	0.22	[-0.25, 0.68]	0.16	4	.26	60.81
Quality of Life	all combined	7 (17)	0.37	[0.18, 0.56]	0.07	5	.004	0

Meta-analytical results for comparing DMI to passive control conditions (where applicable) with outliers removed. Key: DMI, Dance Movement Intervention; k, number of studies; *N* ES, Number of Effect Sizes; ES, Effect size; CI, Confidence interval; SE, Standard Error; df, Degrees of freedom. *Where df <4, p-values are unreliable and are thus not reported here.
